# Disrupted balance of CD4^+^ T-cell subsets in bone marrow of patients with primary immune thrombocytopenia

**DOI:** 10.7150/ijbs.33779

**Published:** 2019-10-23

**Authors:** Qian Wang, Juan Li, Tian-shu Yu, Yu Liu, Kai Li, Shuang Liu, Yang Liu, Qi Feng, Lei Zhang, Guo-sheng Li, Lin-lin Shao, Jun Peng, Ming Hou, Xin-guang Liu

**Affiliations:** 1 Department of Hematology, Qilu Hospital, Shandong University, 107 West Wenhua Road, Jinan, P. R. China;; 2 Department of Clinical Laboratory, Qilu Hospital, Shandong University (Qingdao), 758 Hefei Road, Qingdao, P. R. China;; 3 School of Chemistry and Pharmaceutical Engineering, Qilu University of Technology, 3501 Daxue Road, Jinan, P. R. China;; 4 Department of Radiotherapy, Zhangqiu People's Hospital, 1920 Huiquan Road, Jinan, P. R. China;; 5 Department of Hematology, Taian Central Hospital, Taian, P. R. China;; 6 Department of Orthopedics, Shandong Provincial Qianfoshan Hospital, Shandong University, Jinan, China; 7 Key Laboratory of Cardiovascular Remodeling and Function Research, Chinese Ministry of Education and Chinese Ministry of Health, Jinan, China

**Keywords:** Primary immune thrombocytopenia, T helper cells, regulatory T cells, bone marrow

## Abstract

Disequilibrium of CD4^+^ T-cell subpopulations in peripheral blood (PB) of patients with primary immune thrombocytopenia (ITP) has been well established, whereas the profile of CD4^+^ T-cell subpopulations in bone marrow (BM) remains elusive. In the present study, the frequencies of T helper 22 (Th22), Th17, Th1, Th2, follicular T helper (Tfh) cells and regulatory T cells (Tregs) as well as their effector cytokines in BM and PB from active ITP patients and healthy controls (HCs) were determined. Results showed that the frequencies of Th22, Th17, Th1, and Tfh cells were significantly higher, but Treg number was remarkably lower in BM from ITP patients than from HCs. In the ITP group, it was notable that the numbers of BM Th22, Th17, Th1, Th2, and Tfh cells were significantly elevated compared with the matched PB counterparts, while Treg number in BM was considerably reduced compared with that in PB. In consistence with the BM Th subset pattern, plasma levels of interleukin (IL)-22, IL-17A, and interferon (INF)-γ in BM from ITP patients were significantly increased compared with that from HCs. Therefore, the balance of CD4^+^ T-cell subsets was disrupted in both BM and PB of ITP patients, suggesting that this might play important roles in the pathophysiological process of ITP.

## Introduction

Primary immune thrombocytopenia (ITP) is an acquired organ-specific autoimmune disorder 1, characterized by transient or persistent decrease of the peripheral blood (PB) platelet count to less than 100 × 10^9^/L in the absence of conditions known to cause thrombocytopenia. The overall incidence of ITP ranges from 2.0 to 5.3 per 10^5^ adults each year 2-4. Manifestations of ITP are very heterogeneous. Most of the patients exhibit no symptoms or minimal bruising, while others may have severe bleeding events, such as gastrointestinal hemorrhage, or intracranial hemorrhage. Aside from the severity of thrombocytopenia, additional factors (age, lifestyle, etc.) affect the risk of bleeding in ITP 5.

Traditionally, ITP is regarded as an autoantibody-mediated disease in which platelets are opsonized by glycoprotein-specific autoantibodies and prematurely cleared in the reticuloendothelial system 6. Antiplatelet autoantibody production is subtly regulated by T helper (Th) cells, and enhanced antiplatelet T-cell reactivity has been observed in ITP 7. It is well known that Th subset balance in peripheral blood (PB) of ITP patients is disrupted, and increased numbers of circulating Th1, Th17, Th22 cells, as well as reduced number or function of CD4^+^CD25^+^FoxP3^+^ regulatory T cells (Tregs) has been reported 8-10. In addition, cytotoxic T lymphocyte (CTL)-mediated platelet lysis also contributes to thrombocytopenia in ITP 11. Therefore, the paradigm for the understanding of ITP pathogenesis has skewed toward a T-cell-centered scheme in this decade 12.

The production of platelets is a complex process that involves the commitment of multipotent stem cells to the megakaryocyte (MK) lineage, and the proliferation, maturation and terminal differentiation of MKs. Bone marrow (BM) is a highly cellular and dynamic tissue composed of hematopoietic cells, stromal cells, endothelial cells, and many types of immune cells. The hematopoietic niches, including the osteoblastic niche and the vascular niche, provide the necessary microenvironment for MK maturation and platelet formation 13. A growing body of emerging evidence indicates that the process of thrombopoiesis is impaired in ITP. A shift to a typical morphological feature of immature, less polyploid, and fewer mature platelet-producing megakaryocytes is commonly observed in ITP 14. It has been demonstrated that antiplatelet autoantibodies could suppress the maturation and apoptosis of megakaryocytes, leading to reduced platelet production 15. T cells are important components of BM microenvironments. Elevated number of CD3^+^ T cells has been reported in BM of patients with ITP 16. Moreover, BM CD8^+^ T cells in ITP were shown to be platelet-specific and activated, which could impair the apoptosis of MKs and contribute to decreased platelet production 17. As CD4^+^ T cells are also abundant in BM, their contribution *in situ* is reasonable. However, there are relatively few data regarding the role of BM CD4^+^ T-cell subsets in the development of ITP. In the present study, the profile of BM CD4^+^ T-cell subsets in active ITP patients was determined. We found that the frequencies of Th1, Th17, Th22, and follicular T helper (Tfh) cells were increased, while Treg number was decreased in BM of ITP patients. These results provide new insights into the mechanisms of the underlying immunopathogenic process in ITP.

## Materials and methods

### Patients and controls

Twenty-seven ITP patients with active disease (15 females and 12 males) were enrolled in this study. The median age of patients was 50 years (range 20 - 76 years). Enrollment took place between September 2016 and June 2017 at the Department of Hematology, Qilu Hospital, Shandong University. Patients were diagnosed according to the criteria established by the International Working Group 18, including history, physical examination, complete blood count, and peripheral blood smear examination consistent with ITP. The patients' platelet counts ranged between 3 and 28 × 10^9^/L, with a median count of 10 × 10^9^/L. Cases complicated with diabetes, cardiovascular diseases, pregnancy, activate infection, or connective tissue diseases such as systemic lupus erythematosus (SLE) were excluded. Previous therapy, including rescue, had to be completed at least 6 weeks before enrollment. BM aspiration and biopsy were done in all patients to further exclude other causes of thrombocytopenia such as myelodysplasia syndrome (MDS) and aplastic anemia (AA). Bleeding severity was graded using the ITP-specific Bleeding Assessment Tool (ITP-BAT) 19.

The healthy control (HC) group consisted of 15 healthy adult volunteers (9 females and 6 males, age range 34 - 60 years, median 47 years) who donated their BM for hematopoietic stem cell transplantation. Platelet counts ranged between 240 and 350 × 10^9^/L, with a median count of 324 × 10^9^/L.

Th2 cells, and Tfh cells as well as chemokine receptors including CXCR3, CCR4, CCR6, and CCR10 were determined in 6 active ITP patients and 6 HCs. Immunofluorescence microscopy analyses of different CD4^+^ T-cell subsets was performed in 5 active ITP patients and 5 HCs. The main characteristics of the enrolled patients are presented in **Table 1**.

This study was approved by the Medical Ethical Committees of Qilu Hospital, Shandong University. Informed consent was obtained from all patients and HCs before enrollment in the study in accordance with the Declaration of Helsinki.

### Flow cytometry analysis of BM and peripheral CD4^+^ T-cell subsets

BM aspirates of the posterior superior iliac spine were obtained by experienced physicians. To evaluate peripheral blood dilution, BM aspirate smears were examined simultaneously. Peripheral venous blood was also collected for determination of circulating CD4^+^ T-cell subsets. Levels of intracellular cytokines were measured by flow cytometry in cytokine-producing cells. Briefly, 400 μl of heparinized BM or peripheral whole blood in equal volume of Roswell Park Memorial Institute (RPMI)-1640 were incubated for 4 hours at 37 °C under 5% CO_2_ in the presence of 25 ng/ml phorbol myristate acetate (PMA), 1 μg/ml ionomycin, and 1.7 μg/ml Golgiplug (Monensin; all from Alexis Biochemicals, San Diego, CA, USA). PMA and ionomycin were pharmacological T-cell-activating agents that mimicked signals generated by T-cell receptor (TCR) complex and had the advantage of stimulating T cells of any antigen specificity. Golgiplug could block intracellular transport mechanisms, leading to the accumulation of cytokines in the cells. After incubation, the cells were stained with phycoerythrin (PE)-Cy5-conjugated anti-CD4 monoclonal antibodies (mAbs) at room temperature in the dark for 20 minutes. Then these cells were stained with fluorescein isothiocyanate (FITC)-conjugated anti-interferon (IFN)-γ mAbs, PE-conjugated anti-IL-17 mAbs and allophycocyanin (APC)-conjugated anti-IL22 mAbs after fixation and permeabilization (eBioscience, San Diego, CA, USA). IgGs of the same-species, same-isotype were used as isotype controls. Analysis was performed on a BD FACSCanto II equipped with BD FACSDiva software (BD Biosciences, Franklin Lakes, NJ, USA).

CD4^+^CD25^+^FoxP3^+^ Tregs were determined using the Human Regulatory T cell Staining Kit (eBioscience, San Diego, CA, USA). In brief, 100 μl of heparinized BM or peripheral whole blood were incubated with a cocktail of FITC-conjugated anti-CD4 mAbs and PE-conjugated anti-CD25 mAbs, fixed and permeabilized, and further stained APC-conjugated anti-FoxP3 mAbs. Th2, Tfh cells, and chemokine receptors including CXCR3, CCR4, CCR6, CCR10, were also determined in 6 ITP patients and 6 HCs. Briefly, heparinized BM and PB blood were incubated with PMA, ionomycin, and Golgiplug. Then cells were stained with PerCP-conjugated anti-CD4 mAbs, fixed and permeabilized, and finally stained with FITC-conjugated anti-IFN-γ mAbs and PE-conjugated anti-IL-4 mAbs. For measurement of Tfh cells, peripheral blood mononuclear cells (PBMCS) and BM blood mononuclear cells (BBMCs) were isolated by gradient centrifugation, and stained with FITC-conjugated anti-CD4 mAbs, APC-conjugated anti-CXCR5 mAbs, and PE-conjugated anti-ICOS mAbs. Surface expression of chemokine receptors were presented as median fluorescence intensity (MFI) and were calculated based on the intensity of the cells incubated with appropriate isotype-matched control IgG as a reference. Cells were also analyzed on a BD FACSCanto II equipped with BD FACSDiva software (BD Biosciences, Franklin Lakes, NJ, USA).

Levels of BM Th22, Th17, Th1, Th2, Tfh cells, and Tregs were also determined in smear using multiple channels immunofluorescence staining. The reagents and experimental protocols were described in detail in the **Supplemental Methods**.

### Enzyme-linked immunosorbent assay, real-time PCR, and chemokine Quantibody® Array

BM aspirates and PB were collected into heparin-anticoagulant vacutainer tubes. Plasma was obtained from all subjects by centrifugation and stored at -80 °C for cytokine detection.

Levels of IFN-γ, IL-17A, and IL-22 were measured using commercial enzyme-linked immunosorbent assay (ELISA) kits (eBioscience, San Diego, CA, USA) following the manufacturer's protocols. The lower detection limits for IFN-γ, IL-17A, and IL-22 were 0.99 pg/ml, 15 pg/ml, and 2.7 pg/ml, respectively.

mRNA expression of IL-4 in PBMCs and BBMC was measured by real-time reverse transcription polymerase chain reaction (RT-PCR) according to a previous described method 20. The primers for IL-4 and GAPDH were as follows: IL-4-F, AGCAGTTCCACAGGCACAAG, IL-4-R, TACTCTGGTTGGCTTCCTTCAC; GAPDH-F, GCACCGTCAAGGCTGAGAAC, GAPDH-R, TGGTGAAGACGCCAGTGGA.

Chemokines in BM and PB plasma samples from 7 ITP patients and 4 HCs were determined. As shown in **Supplemental Table 1**, the Quantibody® array (RayBiotech, Norcross, GA, USA) capable of detecting 40 kinds of chemokines/cytokines simultaneously was used according to the manufacturer's instruction.

### The indirect modified monoclonal antibody-specific immobilization of platelet antigens assay

The modified mAb-specific immobilization of platelet antigens (MAIPA) assay was carried out according to a previous described method 21. Briefly, 1 × 10^9^ platelets from healthy donors with blood type O were sensitized with 100 μl plasma from patients or HCs, washed and solubilized in Tris-buffered saline containing 1% Triton X-100 and 0.1 mg/ml leupeptin. Microtiter plates were coated with affinity-purified goat-anti mouse IgG, and incubated with anti-CD41a mAbs or anti-CD42b mAbs (BD Pharmingen, San Jose, CA, USA) for 60 minutes at room temperature. After washing, the sensitized platelet lysate was added in duplicates to each well and incubated for another 60 minutes. IgG bound to captured GPIIb/IIIa or GPIb/IX was detected by alkaline-phosphatase-conjugated goat anti-human IgG. p-Nitrophenyl-phosphate was used as the substrate and the plates were read on an automated microtiter plate reader (Thermo-Multiskan Mk3; Hudson, NH, USA) using dual wavelength (405 and 492 nm). A positive result was defined as absorbance above mean + 3 standard deviations (SDs) of normal controls.

### Statistical analysis

Statistical analysis was performed using SPSS 19.0 software. All continuous values were expressed as means ± standard deviation (SD). Descriptive statistics were used to summarize demographic and baseline clinical characteristics of the enrolled patients. Statistical difference between ITP patients and HCs was determined by independent sample *t* test unless the data were not normally distributed, in which case the Mann-Whitney *U* test was used. Comparisons of absolute values between BM and PB in ITP patients or HCs were made using the paired Student *t* test. Pearson correlation test was used for correlation analysis depending on data distribution. *P* values < 0.05 were considered statistically significant.

## Results

### Elevated levels of Th22 cells and IL-22 in the BM and PB of ITP patients

**Figure 1:**
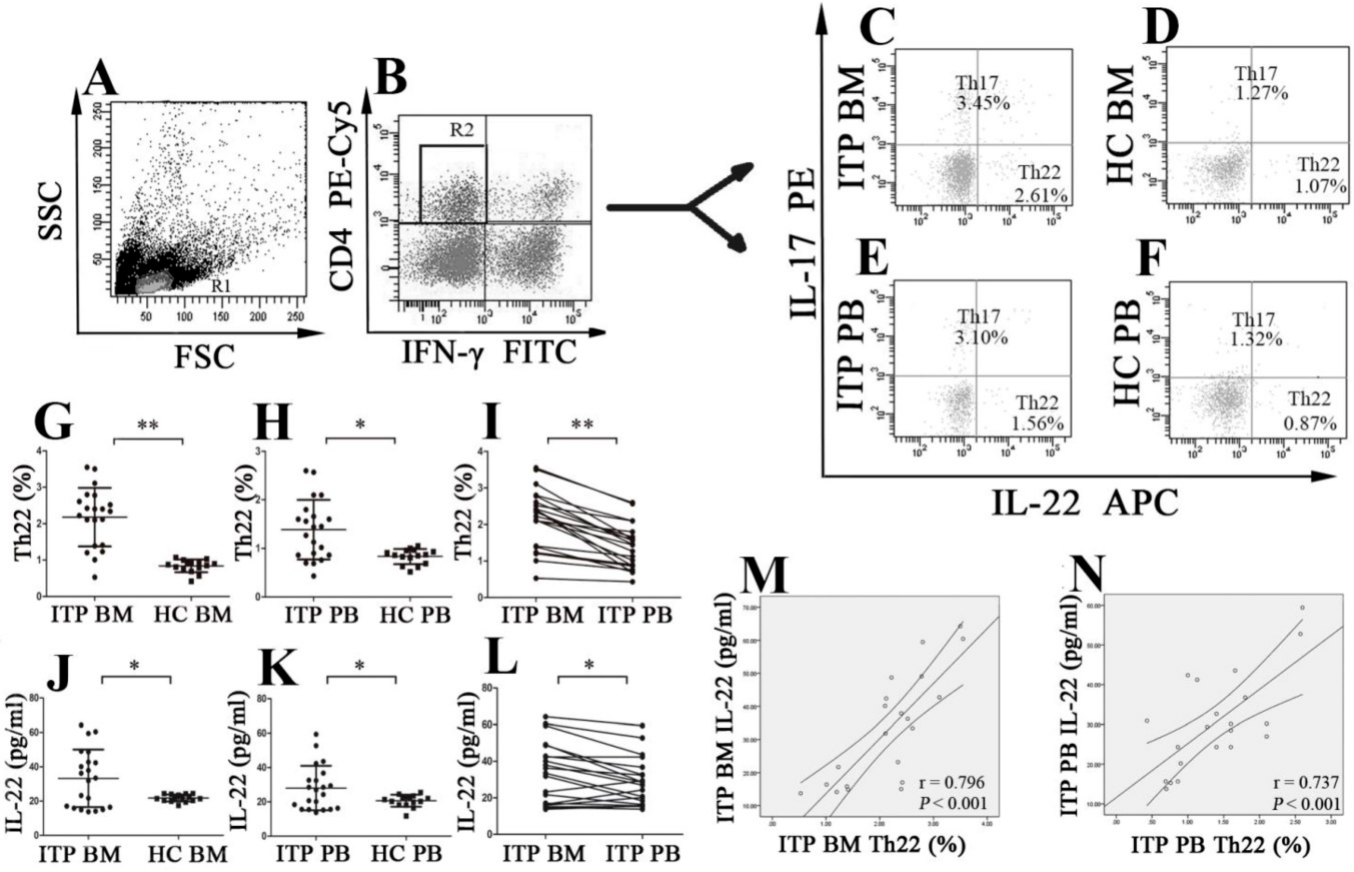
** The percentages of Th22 cells and plasma levels of IL-22 in BM and PB from ITP patients and healthy controls (HCs).** Heparinized BM and PB from all subjects were stained with labeled antibodies and analyzed by flow cytometry. **(A)** Lymphocytes were gated based on their forward and side scatter. **(B)** CD4^+^IFN-γ^-^ lymphocytes were further gated for analysis of Th22 and Th17 cells. **(C, D, E, F)** Representative scattergrams of Th22 and Th17 cells in BM and PB from ITP patients and HCs. **(G, H, J, K)** Th22 cells and IL-22 in BM and PB from ITP patients were significantly higher than their matched BM and PB counterparts from HCs. **(I, L)** In ITP group, the percentage of BM Th22 cells and plasma level of IL-22 were remarkably higher than the PB counterparts. **(M, N)** Plasma levels of IL-22 correlated positively with the percentages of Th22 cells both in BM and PB in ITP group. Bars represent means ± SD. * *P* < 0.05, ** P < 0.001.

BM aspirate smears were performed for all enrolled patients and HCs, and peripheral blood dilution in the BM was not observed in any of the included subjects. Frequencies of different CD4^+^ T-cell subsets were analyzed based on cytokine patterns after *in vitro* activation by PMA/ionomycin. The cells were gated by forward and side scatter for lymphocytes (**Figure 1A**), and then CD4^+^IFN-γ^-^ T cells (**Figure 1B**) were identified for analysis of Th17 and Th22 cells. Th22 subset was defined as CD4^+^IL22^+^IFNγ^-^IL17^-^ T cells thereby excluding Th1 and Th17 cells. The typical dot plots of BM and PB Th22 cells in ITP patients and HCs were shown in **Figure 1C, D, E** and** F**. The percentage of BM Th22 cells from ITP patients was significantly higher than from HCs (2.18 ± 0.80% *vs.* 0.84 ± 0.17%, *P* < 0.001;** Figure 1G**). Immunofluorescence microscopy also revealed that the percentage of BM Th22 cells was higher from ITP patients than from HCs, but this difference did not achieve statistical significance (*P* = 0.082; **Supplemental Figure 1 A, B** and**Supplemental Figure 2A**). The discrepancy might be due to the greater sensitivity of flow cytometry when compared to immunofluorescence microscopy. In line with the BM Th22 pattern, frequency of PB Th22 cells from ITP patients was also remarkably higher compared to HCs (1.39 ± 0.61% *vs.* 0.83 ± 0.16%; *P* = 0.001; **Figure 1H**). In the ITP group, it was notable that the percentage of BM Th22 cells was significantly elevated than the paired PB Th22 cells (2.18 ± 0.80% *vs.* 1.39 ± 0.61%; *P* < 0.001; **Figure 1I**). With regard to the HCs, BM Th22 cells showed no statistical difference compared to their PB counterparts (0.84 ± 0.17 *vs.* 0.83 ± 0.16%, *P* = 0.870).

Plasma IL-22 concentrations of BM and PB were measured by ELISA. Level of BM IL-22 from ITP patients was significantly higher than from HCs (33.26 ± 16.77 *vs.* 21.80 ± 2.06 pg/ml, *P* = 0.005;** Figure 1J**). Consistent with our previous reports 22, plasma level of IL-22 in PB from ITP patients was also considerably increased in comparison with that from HCs (28.04 ± 12.96 *vs.* 20.67 ± 3.49 pg/ml, *P* = 0.020; **Figure 1K**). Moreover, in the ITP group, level of BM IL-22 was significantly elevated compared with that of the paired PB IL-22 (33.26 ± 16.77 *vs.* 28.04 ± 12.96 pg/ml, *P* = 0.007; **Figure 1L**). By contrast, no statistical difference was found in plasma IL-22 level between BM and PB in HCs (21.80 ± 2.06 *vs.* 20.67 ± 3.49 pg/ml; *P* = 0.360). In ITP patients, positive correlations were found between the frequency of Th22 cells and IL-22 level both in BM and in PB (BM: *r* = 0.796, *P* < 0.001; PB: *r* = 0.737, *P* < 0.001, respectively; **Figure 1M** and** N**).

### Skewed balance of Th17/Treg in the BM and PB of ITP patients

**Figure 2:**
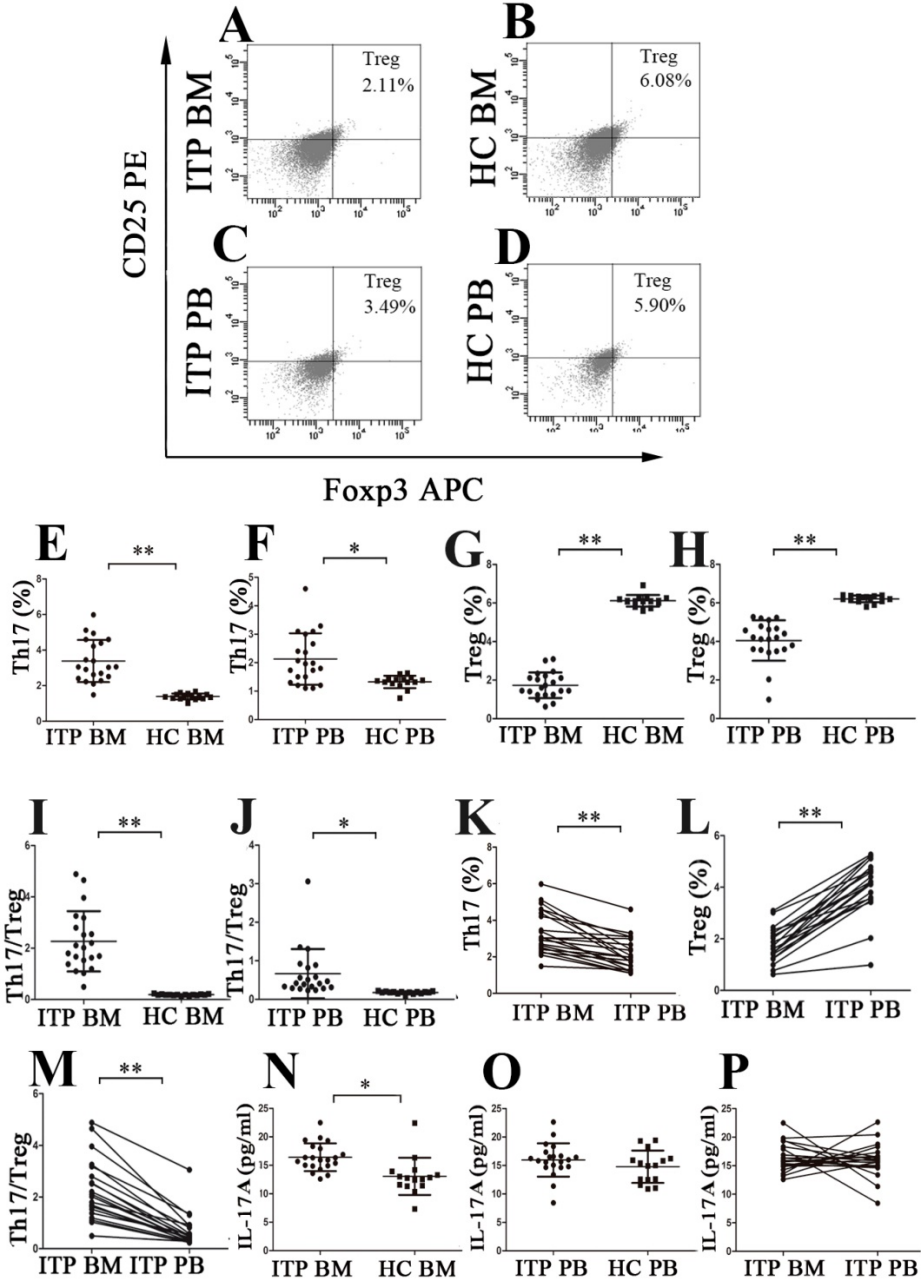
** The percentages of Th17 cells and Tregs as well as plasma levels of IL-17 in BM and PB from ITP patients and HCs. (A, B, C, D)** Representative scattergrams of Tregs in BM and PB from ITP patients and HCs. **(E, F, G, H)** The percentages of Th17 cells were significantly higher, while Tregs were remarkably lower than their matched BM and PB counterparts from HCs. **(I, J)** BM and PB Th17/Treg ratios were markedly elevated from ITP patients compared to their counterparts from HCs. **(K, L)** In ITP group, the percentage of BM Th17 cells was remarkably higher, while Tregs was considerably lower than the PB counterparts. **(M)** BM Th17/Treg ratio was considerably higher than the PB counterparts from ITP patients. **(N)** Plasma level of IL-17A in BM from ITP patients was significantly higher than from HCs. **(O)** There was no statistical difference in plasma PB IL-17A level between ITP patients and HCs. **(P)** In ITP group, no statistical difference was found in plasma levels of IL-17A between BM and PB. Bars represent means ± SD. * *P* < 0.05, ** P < 0.001.

Th17 cells and Tregs were analyzed using the well-established gating strategy. The population of CD4^+^IFN-γ^-^IL17^+^ T cells was identified as Th17 subset (**Figure 1C, D, E** and** F**), and Tregs were defined as CD4^+^CD25^+^FoxP3^+^ T cells (**Figure 2A, B, C** and** D**). Results showed that the percentage of BM Th17 cells from ITP patients was significantly higher than from HCs (3.38 ± 1.18% *vs.* 1.39 ± 0.17%, *P* < 0.001; **Figure 2E**), and the level of PB Th17 cells from ITP patients was also remarkably increased compared to HCs (2.13 ± 0.90% *vs.* 1.32 ± 0.22%, *P* = 0.001; **Figure 2F**). Moreover, BM Th17 cells determined by immunofluorescence microscopy were also higher from ITP patients compared with HCs but statistical significance was not reached (*P* = 0.190; **Supplemental Figure 1 C, D** and**Supplemental Figure 2B**). By contrast, the frequencies of BM Tregs determined by flow cytometry or immunofluorescence microscopy were considerably lower from ITP patients than from HCs (flow cytometry: 1.73 ± 0.66% *vs.* 6.12 ± 0.30%, *P* < 0.001;** Figure 2G;** immunofluorescence microscopy:* P* = 0.015; **Supplemental Figure 1I, J** and**Supplemental Figure 2C**), and the percentage of PB Tregs from ITP patients was also reduced in comparison with that from HCs. (4.05 ± 1.05% *vs.* 6.21 ± 0.18%, *P* < 0.001;** Figure 2H**). As a result, the ratio of Th17 cells to Tregs in BM and PB from ITP patients was significantly higher than from HCs (BM: 2.27 ± 1.18 *vs.* 0.19 ± 0.03, *P* < 0.001; PB: 0.67 ± 0.64 *vs.* 0.18 ± 0.03, *P* = 0.006, respectively;** Figure 2I** and** J**). Interestingly, significantly increased level of Th17 and decreased level of Tregs in BM were observed compared with those in PB from ITP patients (Th17: 3.38 ± 1.18% *vs.* 2.13 ± 0.90%; *P* < 0.001; Tregs: 1.73 ± 0.66% *vs.* 4.05 ± 1.05%, *P* < 0.001, respectively;** Figure 2K** and** Figure 2L**). Therefore, the ratio of Th17 cells to Tregs was higher in BM than in PB from ITP patients (2.27 ± 1.18 *vs.* 0.67 ± 0.64, *P* < 0.001;** Figure 2M**). With respect to HCs, there was no statistical difference in frequency of Th17 cells between BM and PB (1.39 ± 0.17% *vs.* 1.32 ± 0.22%, *P* = 0.304), nor for Tregs frequency between BM and PB (6.12 ± 0.30% *vs.* 6.21 ± 0.18%, *P* = 0.354).

The level of BM IL-17A from ITP patients was higher than from HCs (16.41 ± 2.43 *vs.*13.05 ± 3.27 pg/ml, *P* = 0.001;** Figure 2N**). Moreover, the PB IL-17A level from ITP patients also showed a slight increase in comparison with that from HCs, but this increase did not achieve statistical significance (15.96 ± 2.93 *vs.*14.77 ± 2.85 pg/ml, *P* = 0.232; **Figure 2O**). We did not observe any statistical difference in IL-17A levels between BM and PB from ITP patients or HCs (ITP: 16.41 ± 2.43 *vs.*15.96 ± 2.93 pg/ml, *P* = 0.658; **Figure 2P**; HCs: 13.05 ± 3.27 *vs.*14.77 ± 2.85 pg/mL, *P* = 0.126).

There was no statistical correlation between the frequency of Th17 cells and IL-17A level in BM from ITP patients (*P* = 0.630). Additionally, frequencies of Th17 cells in PB also failed to show any statistical correlation with plasma level of IL-17A in PB from ITP patients (*P* = 0.281). There was no significant correlation between levels of IL-17 and IL-22 or IFN-γ in BM, nor between levels of IL-17 and IL-22 or IFN-γ in PB from ITP patients (all *P >* 0.05).

### Increased expression of Th1 cells in the BM and PB of ITP patients

**Figure 3:**
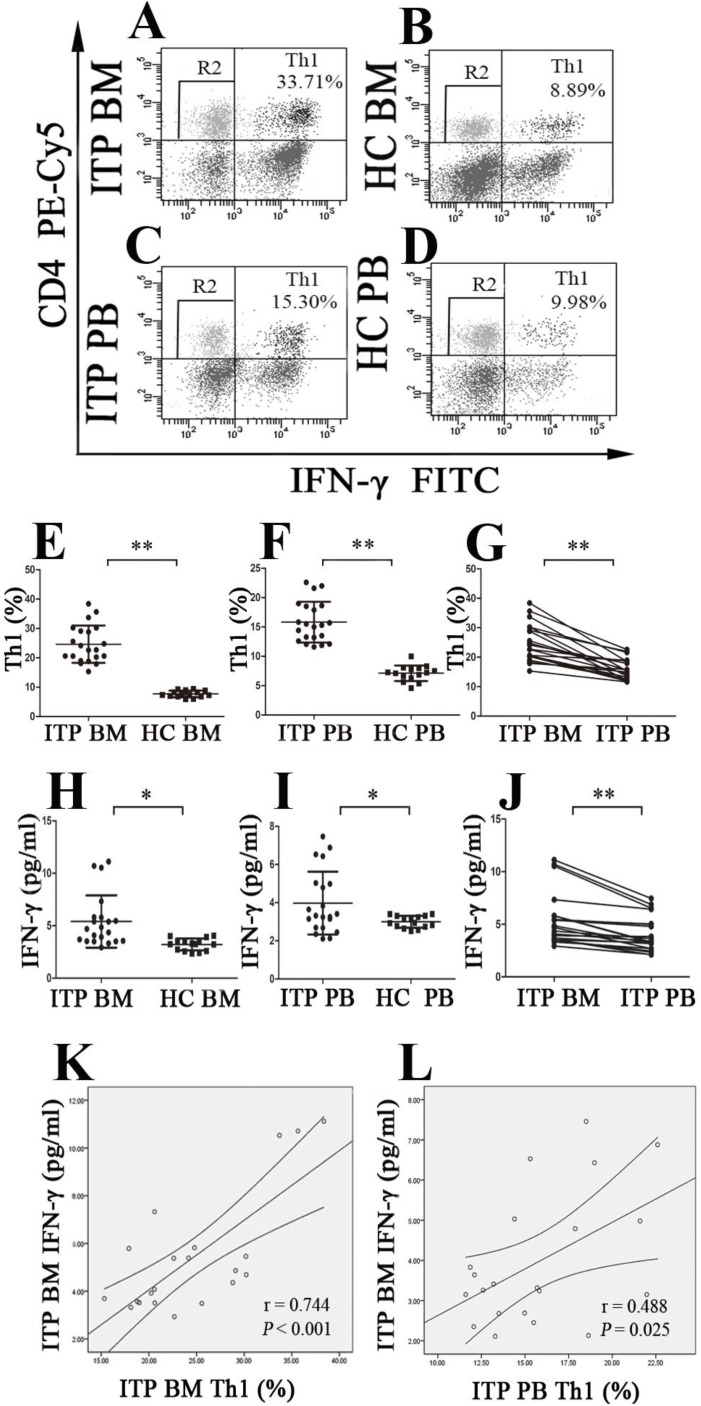
** BM and PB Th1 cells and INF-γ from ITP patients and HCs. (A, B, C, D)** Representative scattergrams of Th1 cells in BM and PB from ITP patients and HCs. **(E, F, H, I)** Th1 cells and INF-γ in BM and PB from ITP patients were significantly higher than their matched BM and PB counterparts from HCs. **(G, J)** In ITP group, BM Th1 cells and INF-γ were remarkably higher than the PB counterparts. **(K, L)** Plasma levels of INF-γ correlated positively with the percentages of Th1 cells both in BM and PB in ITP group. Bars represent means ± SD. * *P* < 0.05, ** P < 0.001.

The population of CD4^+^IFN-γ^-+^ T cells was identified as Th1 cells (**Figure 3A, B, C** and **D**). As demonstrated in** Figure 3E**, the portion of BM Th1 cells from ITP patients was remarkably increased compared to HCs (24.62 ± 6.37% *vs.* 7.70 ± 1.12%, *P <* 0.001), and the percentage of PB Th1 cells in ITP group was also significantly higher than in HCs (15.81 ± 3.47% *vs.* 7.11 ± 1.33%, *P* < 0.001; **Figure 3F**). Consistently, BM Th1 cells determined by immunofluorescence microscopy were marked elevated from ITP patients compared with HCs (*P* = 0.048; **Supplemental Figure 1 E, F, G, H** and**Supplemental Figure 2D**). We also observed that the percentage of Th1 cells in BM was significantly increased in comparison with PB from ITP patients (24.62 ± 6.37% *vs.* 15.81 ± 3.47%, *P* < 0.001; **Figure 3G**), yet no statistical difference was found in the portion of Th1 cells between BM and PB from HCs (7.70 ± 1.128% *vs.* 7.11 ± 1.33%, *P =* 0.083).

Plasms IFN-γ levels in BM and PB from enrolled subjects were also evaluated. The data showed that BM IFN-γ concentration from ITP patients was significantly higher than from HC (5.40 ± 2.50 *vs.* 3.21 ± 0.57 pg/ml, *P* =0.001; **Figure 3H**), and PB IFN-γ concentration from ITP patients and HCs showed a similar pattern (3.98 ± 1.65 *vs.* 3.00 ± 0.31 pg/ml, *P* = 0.014; **Figure 3I**). Compared to the IFN-γ level in PB from ITP patients, the paired BM IFN-γ level was remarkably increased (3.98 ± 1.65 *vs.* 5.40 ± 2.50 pg/ml, *P* < 0.001; **Figure 3J**). With regard to HCs, there was no statistical difference in IFN-γ level between BM and PB (3.21 ± 0.57 *vs.* 3.00 ± 0.31 pg/ml, *P* = 0.209). In ITP patients, percentage of Th1 cells correlated positively with plasma level of IFN-γ in both BM and PB (BM: *r* = 0.744, *P* < 0.001; PB: *r* = 0.488, *P* = 0.025, respectively; **Figure 3K** and** L**).

### Expression of Th2 and Tfh cells in the BM and PB of ITP patients

**Figure 4:**
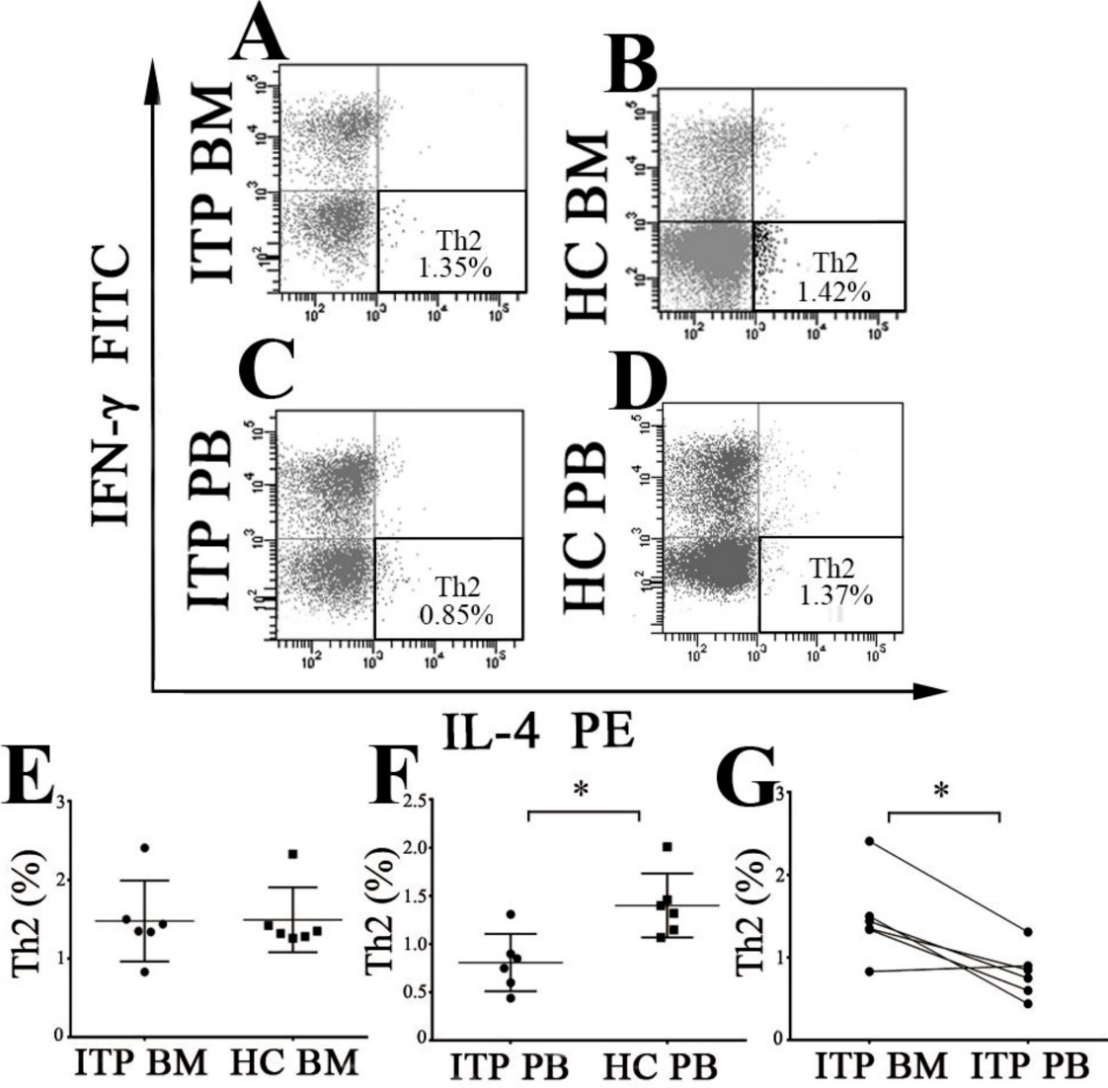
** BM and PB Th2 cells from ITP patients and HCs. (A, B, C, D)** Representative scattergrams of Th2 cells in BM and PB from ITP patients and HCs. **(E)** There was no statistical difference in BM Th2 cells between ITP patients and HCs. **(F)** Th2 cells in PB from ITP patients was significantly lower compared with HCs. **(G)** In ITP group, frequency of BM Th2 cells was remarkably higher than the PB counterparts. Bars represent means ± SD. * *P* < 0.05.

Th2 subset was identified as CD4^+^IL-4^+^IFN-γ^-^ T cells (**Figure 4A, B, C** and** D**). As shown in **Figure 4E**, there was no statistical difference in BM Th2 frequency between ITP patients and HCs when measured by flow cytometry (1.47 ± 0.51 % *vs.* 1.49 ± 0.41 %, *P* = 0.949) or immunofluorescence microscopy (*P* = 0.692; **Supplemental Figure 1 C, D** and**Supplemental Figure 2E).** On the contrary, PB Th2 frequency from ITP patients was significantly lower than from HCs (0.81 ± 0.30 % *vs.* 1.40 ± 0.33 %, *P* = 0.007; **Figure 4F**). In the ITP group, BM Th2 percentage was remarkably higher than the pair PB counterpart (1.47 ± 0.51 % *vs.* 0.81 ± 0.30%, *P* = 0.012; **Figure 4G**). By contrast, there was no statistical difference in Th2 frequency between BM and PB in HCs (1.49 ± 0.41 % *vs.* 1.40 ± 0.33 %, *P* = 0.721).

**Figure 5:**
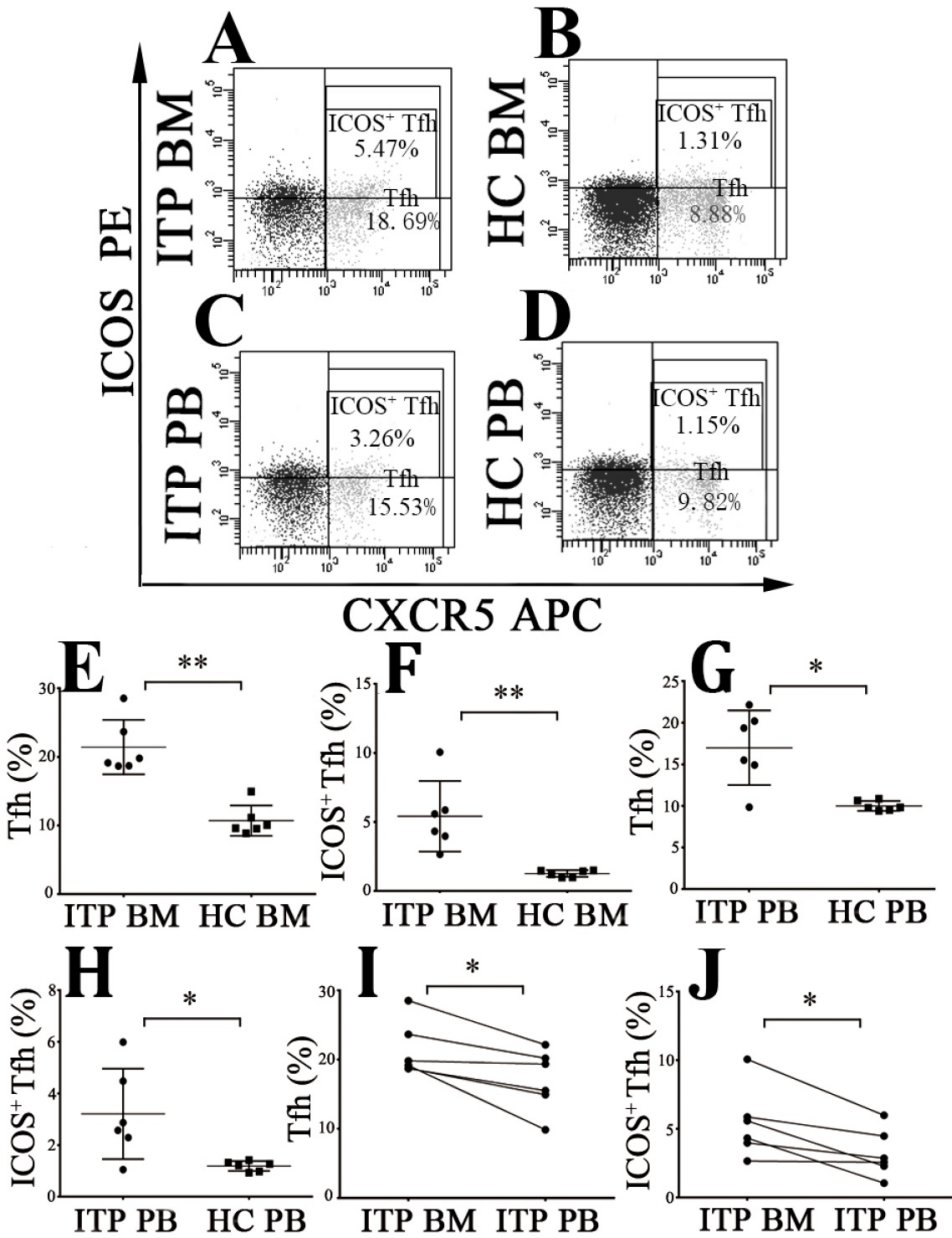
** Tfh and ICOS^+^ Tfh cells in BM and PB from ITP patients and HCs. (A, B, C, D)** Representative scattergrams of Tfh and ICOS^+^ Tfh cells in BM and PB from ITP patients and HCs. **(E, F, G, H)** Tfh and ICOS^+^ Tfh cells in BM and PB from ITP patients were significantly higher than their matched BM and PB counterparts from HCs. **(I, J)** In ITP group, BM Tfh and ICOS^+^ Tfh cells were remarkably higher than the PB counterparts. Bars represent means ± SD. * *P* < 0.05, ** P < 0.001.

The frequencies of CD4^+^CXCR5^+^ Tfh cells and CD4^+^CXCR5^+^ICOS^+^ Tfh cells were measured (**Figure 5A, B, C** and** D**). We found that BM CD4^+^CXCR5^+^ and CD4^+^CXCR5^+^ICOS^+^ Tfh levels from ITP patients were considerably increased than from HCs (CD4^+^CXCR5^+^: 21.43 ± 3.94 % *vs.* 10.71 ± 2.21 %, *P* < 0.001; CD4^+^CXCR5^+^ICOS^+^: 5.42 ± 2.56 % *vs.* 1.26 ± 0.24 %, *P* < 0.001; **Figure 5E** and **F**). Consistently, PB CD4^+^CXCR5^+^ Tfh and CD4^+^CXCR5^+^ICOS^+^ Tfh levels from ITP patients were also higher than from HCs (CD4^+^CXCR5^+^: 17.01 ± 4.47 % *vs.* 10.01 ± 0.60 %, *P* = 0.001; CD4^+^CXCR5^+^ICOS^+^: 3.21 ± 1.75 % *vs.* 1.18 ± 0.19 %, *P* = 0.036; **Figure 5G** and** H**). Moreover, BM CD4^+^CXCR5^+^ Tfh and CD4^+^CXCR5^+^ICOS^+^ Tfh percentages were significantly higher than their PB counterparts in ITP group (CD4^+^CXCR5^+^: 21.43 ± 3.94 % % *vs.* 17.01 ± 4.47 %, *P* = 0.016; CD4^+^CXCR5^+^ICOS^+^: 5.42 ± 2.56 % *vs.* 3.21 ± 1.75 %, *P* = 0.018; **Figure 5I** and** J**). No statistical significance was found in CD4^+^CXCR5^+^ Tfh or CD4^+^CXCR5^+^ICOS^+^ Tfh percentages between BM and PB in HCs (CD4^+^CXCR5^+^: 10.71 ± 2.21 % *vs.* 10.01 ± 0.60 %, *P* = 0.499; CD4^+^CXCR5^+^ICOS^+^: 1.26 ± 0.24 % *vs.* 1.18 ± 0.19 %, *P* = 0.465).

mRNA expression of IL-4, the key cytokine of Th2 cells, was also determined using real-time RT-PCR. It was observed that PB IL-4 mRNA level from ITP patients was significantly lower than from HCs (0.000206 ± 0.000038 *vs*. 0.00033 ± 0.000071, *P* = 0.017), while no statistical difference was observed in BM IL-4 mRNA level between ITP patient and HCs (0.000345 ± 0.000107 *vs*. 0.000369 ± 0.000099, *P* = 0.630). In the ITP group, BM IL-4 mRNA level was considerably increased compared to its PB counterpart (0.000345 ± 0.000107 *vs*. 0.000206 ± 0.000038, *P* = 0.012). We did not find any statistical difference in IL-4 mRNA level between BM and PB in the HC group (0.000369 ± 0.000099 *vs*. 0.00033 ± 0.000071, *P* = 0.354).

### Association of different CD4^+^ T-cell subsets in BM and PB with disease duration, previous treatments, and bleeding severity in ITP patients

Subgroup analyses were performed to explore whether the aberrant CD4^+^ T-cell distribution was related to disease duration, previous treatments, or bleeding severity. As shown in **Table 2, 3** and** 4**, there was no statistical difference in BM or PB levels of Th22, Th17, Th1 cells, Tregs, and Th17/Treg ratios as well as their signature cytokines between newly diagnosed/persistent and chronic ITP patients, nor between treatment-naive and recurrent ITP patients. With respect to bleeding severity, frequency of PB Th22 cells was significantly higher from bleeding grade 2 or 3 patients than from bleeding grade 1 patients (1.78 ± 0.50% *vs*. 1.31 ± 0.53 %, *P* = 0.017). Level of BM Th22 cells from bleeding grade 2 or 3 patients was also elevated compared with that from bleeding grade 1 patients, but this elevation did not achieve statistical significance (2.54 ± 0.65 % *vs*. 2.03 ± 0.72 %, *P* = 0.053). In addition, we also observed that frequency of BM Th1 cells from bleeding grade 2 or 3 patients was remarkably increased than from bleeding grade 1 patients (30.28 ± 6.72 % *vs*. 24.19± 6.00 %, *P* = 0.006), whereas there was no statistical difference in level of PB Th1 cells between grade 2 or 3 patients and grade 1 patients (19.13 ± 7.94 % *vs*. 17.37 ± 5.02 %, *P* = 0.404). We did not observe any statistical difference in BM and PB Th17 cells, Tregs, and Th17/Treg ratios between bleeding grade 2 or 3 patients and bleeding grade 1 patients (all* P >* 0.05, **Table 5**).

### Chemokine receptor expression on different CD4^+^ T-cell subsets and chemokine profile in BM and PB of ITP patients and HCs

**Figure 6:**
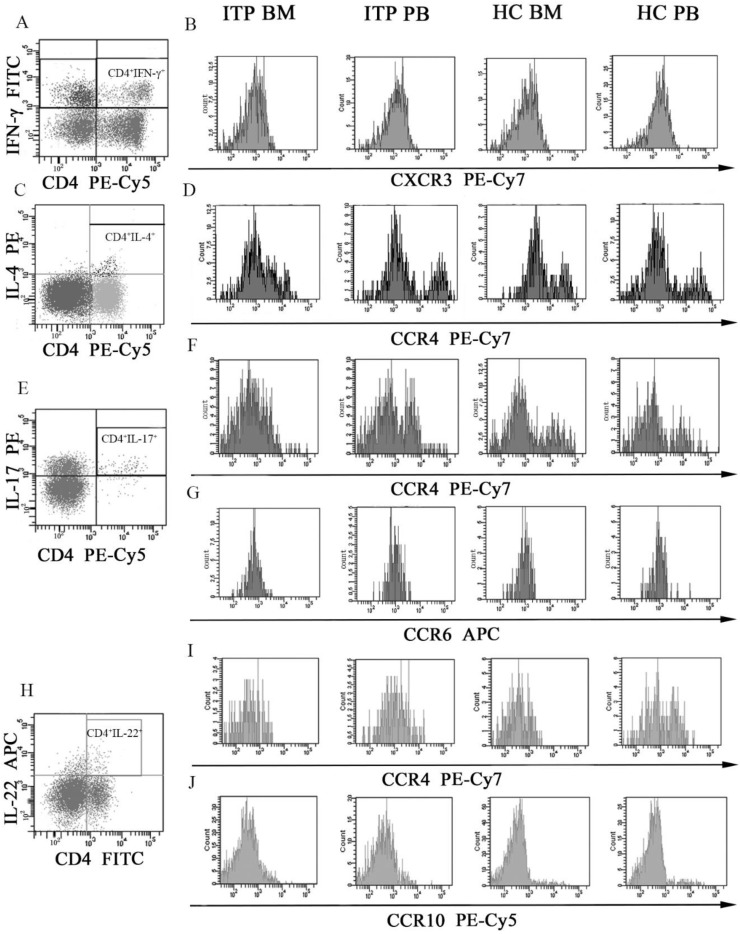
** MFI histogram of Chemokine receptors on different CD4^+^ T-cell subsets in BM and PB from ITP patients and HCs. (A, B)** Representative MFI histogram of CXCR3 on CD4^+^IFN-γ^+^ T cells in BM and PB from ITP patients and HCs. **(C, D)** Representative MFI histogram of CCR4 on CD4^+^IL-4^+^ T cells in BM and PB from ITP patients and HCs. **(E, F, G)** Representative MFI histogram of CCR4 and CCR6 on CD4^+^IL-17^+^ T cells in BM and PB from ITP patients and HCs. **(H, I, J)** Representative MFI histogram of CCR4 and CCR10 on CD4^+^IL-22^+^ T cells in BM and PB from ITP patients and HCs.

Several chemokine receptors which were critical for migration and differentiation of different CD4^+^ T-cell subsets were determined by flow cytometry. MFI of CXCR3 on CD4^+^IFN-γ^+^ T cells (**Figure 6A** and **B**), CCR4 on CD4^+^IL-4^+^ T cells (**Figure 6C** and **D**), CCR4 and CCR6 on CD4^+^IL-17^+^ T cells (**Figure 6E, F** and **G**), and CCR4 and CCR10 on CD4^+^IL-22^+^ T cells (**Figure 6H, I** and **J**) were assessed. As shown in **Table 6**, CXCR3 levels on BM and PB CD4^+^IFN-γ^+^ T cells from ITP patients were significantly higher than from HCs (BM: 1527.3 ± 216.1 *vs*. 1063.5 ± 217.5, *P* = 0.001; PB: 1705.5 ± 235.1 *vs*. 1293.7 ± 141.6, *P* = 0.002). In the ITP group, CD4^+^IFN-γ^+^ T cells in BM expressed lower level of CXCR3 than in PB (1527.3 ± 216.1 *vs*. 1705.5 ± 235.1, *P* = 0.033). Moreover, CXCR3 level on BM CD4^+^IFN-γ^+^ T cells was also decreased comparted with its PB counterpart in HCs (1063.5 ± 217.5 *vs*. 1293.7 ± 141.6, *P* = 0.009).

CCR4 levels on BM and PB CD4^+^IL-22^+^ T cells from ITP patients were remarkably elevated compared with HCs (BM: 2584.3 ± 824.5 *vs*. 1624.8 ± 217.4, *P* = 0.005; PB: 2402.8 ± 607.3 *vs*. 1496.2 ± 155.4, *P* = 0.008). By contrast, CCR4 on CD4^+^IL-4^+^ and CD4^+^IL-17^+^ T cells, CCR6 on CD4^+^IL-17^+^ T cells, CCR10 on CD4^+^IL-22^+^ T cells, and CXCR4 on CD4^+^FoxP3^+^ T cells in BM or PB showed no statistical difference between ITP patients and HCs (all* P >* 0.05).

**Figure 7:**
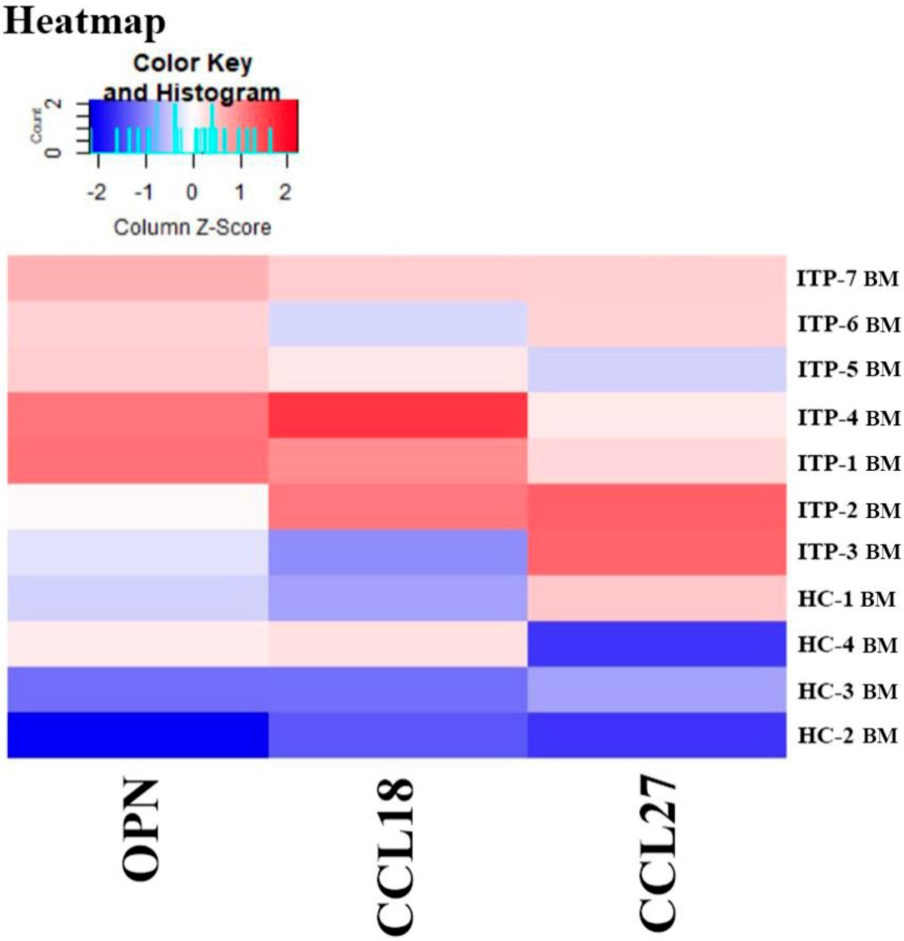
Heatmap of differential expressed proteins between ITP BM and HC BM.

**Figure 8:**
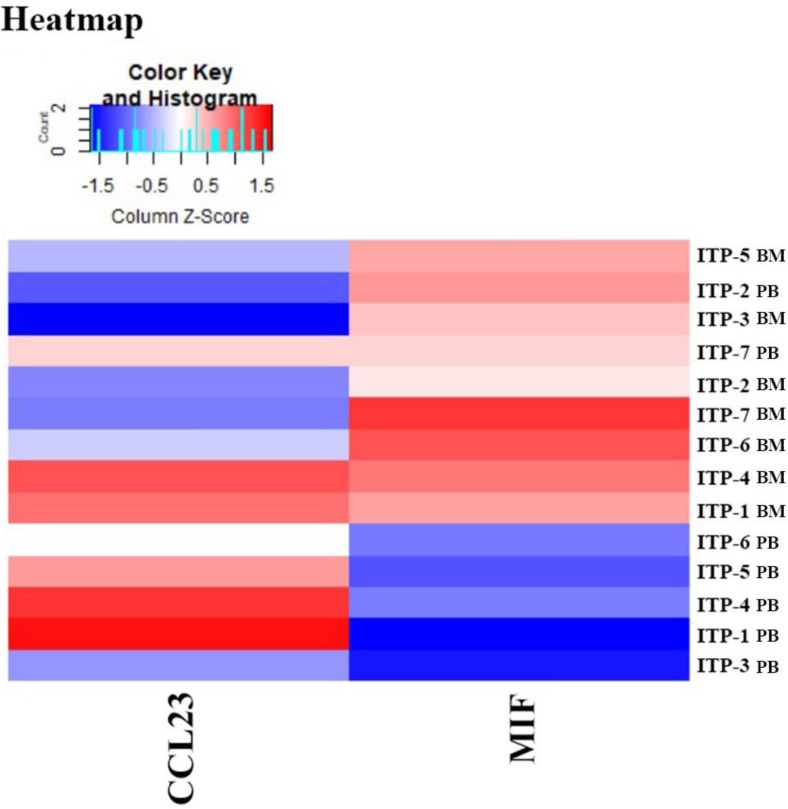
Heatmap of differential expressed proteins between ITP BM and ITP PB.

Results of the Quantibody® array showed that BM levels of CCL27, osteopontin (OPN), and CCL18 from ITP patients were significantly higher than from HCs (CCL27: 3218.39 *vs*. 1190.38, *P* = 0.041; OPN: 8175.97 vs. 2293.69, *P* = 0.016; CCL18: 807.64 *vs*. 628.95, *P* = 0.039; **Figure 7**). By contrast, the 40 kinds of chemokines/cytokines in PB showed no statistical difference between ITP patients and HCs. In the ITP group, BM levels of macrophage migration inhibitory factor (MIF) was considerably higher, while CCL23 was remarkably lower than their PB counterparts (MIF: 11814.48 *vs*. 2412.42, *P* = 0.025; CCL23: 590.24 *vs*. 745.20, *P* = 0.018; **Figure 8**).

### Correlations of every different CD4^+^ T-cell subset between BM and PB in ITP patients

**Figure 9:**
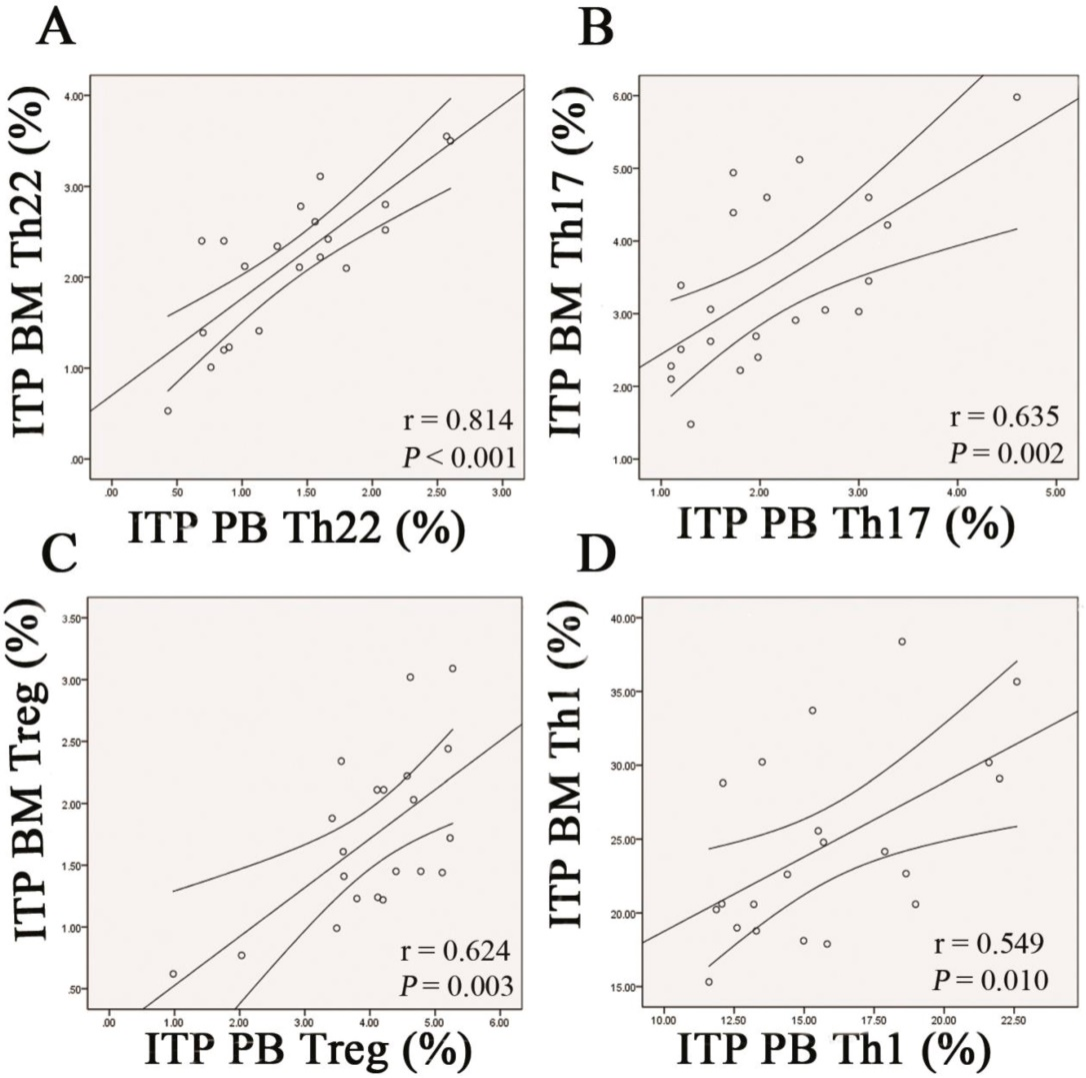
** Correlations of each CD4^+^ T-cell subpopulation between BM and PB from ITP patients.** Pearson correlation analysis revealed that the percentages of BM Th22, Th17, Th1 cells, and Tregs correlated positively with their PB counterparts in ITP group.

Pearson correlation test was performed to evaluate the correlations between BM and PB for the frequencies of each CD4^+^ T subpopulation in ITP patients. The data demonstrated that frequency of Th22 cells in BM was positively correlated with that in PB (*r* = 0.814, *P* < 0.001; **Figure 9A**). Likewise, frequencies of Th17 cells and Tregs in BM were positively correlated with those in PB (Th17: *r* =0.635, *P* = 0.002; Treg: *r* = 0.624, *P* = 0.002; **Figure 9B** and** C**). With respect to Th1 subset, its BM frequency also showed positive correlation with the PB counterpart in ITP patients (*r* = 0.549, *P* = 0.010; **Figure 9D**).

### Correlations of different CD4^+^ T-cell subsets in BM and PB with autoantibodies in ITP patients

To evaluate whether the altered CD4^+^ T-cell profile was associated with platelet GP-specific autoantibody production, plasma GPIIb/IIIa and GPIb/IX autoantibodies were determined. Results showed that there was no significant difference in the frequencies of BM Th22, Th17, Th1 cells and Tregs between ITP patients with positive autoantibodies and those with negative results (all *P >* 0.05), nor for the frequencies of PB Th22, Th17, Th1 cells, and Tregs between antibody-positive patients and antibody-negative patients (all *P >* 0.05; **Supplemental Table 2**).

## Discussion

T cells still take the center-stage in the immunopathogenesis of ITP by initiating, propagating and maintaining the antiplatelet autoimmunity 12. Peripheral tolerance defects of CD4^+^ T cells in ITP have been attributed to enhanced antiplatelet T-cell reactivity 7, resistance of autoreactive T cells to activation induced cell death (AICD) 23, increased numbers of Th1, Th17, Th22 cells, and reduced number or function of Tregs 10,11. Thrombopoiesis, which occurs from megakaryocytes in the BM, has been shown to be impaired in ITP 15. So far, relatively little is known about the profile of CD4^+^ T cells in BM of ITP patients. We investigated the levels of different CD4^+^ T-cell subpopulations, and found that significantly elevated numbers of Th1, Th17, and Th22 cells coincided with considerably decreased number of Tregs in BM of active ITP patients, suggesting dysregulated immune responses might take place in the BM microenvironment.

Th22 subset is a more recently identified CD4^+^ Th subpopulation characterized by secretion of IL-22 but not IL-17 or IFN-γ 24,25. It is a terminally differentiated T-cell subtype and can be induced from naïve T cells in the presence of tumor necrosis factor (TNF)-α and IL-6 24. Growing bodies of emerging evidence have indicated that Th22 cells were involved in the pathogenesis of a variety of autoimmune diseases in humans, such as SLE 26, rheumatoid arthritis (RA) 27, psoriasis 28, and Crohn's disease 29. In PB of ITP patients, our group along with several others reported consistently that frequency of Th22 cells was significantly increased 9,30. Consistently, our present study further confirmed the elevated level of PB Th22 cells in ITP patients. Of note, we demonstrated for the first time that level of BM Th22 cells and IL-22 was even higher than their PB counterparts in ITP patients. Up to now, the mechanism through which these upregulated Th22 cells take part in the pathophysiological process of ITP still remains to be elucidated. As the receptor of IL-22 is only expressed on epithelial and stromal cells instead of immune cells 31, IL-22 produced by Th22 cells might exacerbate the immune dysregulation of ITP through unknown indirect mechanisms. Recently, Muñoz et al. demonstrated that IL-22 promoted the secretion of IL-18 from epithelial cells during intestinal infection 32. With regard to ITP, our published data have established the pathogenetic role of IL-18 in Th1 polarization 33. Therefore, IL-22-mediated enhancement of Th1 response through IL-18 upregulation might be possible. Aside from IL-22, Th22 cells also produce TNF-α to some extent, which might play a role in macrophage activation and platelet destruction in ITP 34.

Th17 cells have strong proinflammatory abilities and play important roles in a variety of inflammatory and autoimmune diseases 35. On the contrary, CD4^+^CD25^+^FoxP3^+^ Tregs shed suppression on the activation and proliferation of T effector cells by cell-to cell contact and secretion of anti-inflammatory cytokines such as IL-10 and transform growth factor (TGF)-β 36. Both Th17 cells and Tregs can develop from naïve CD4^+^ T cells under the influence of the same cytokine, TGF-β1 37, whereas accumulating evidence indicates that Th17 cells and Tregs functionally antagonize each other 38-40. In patients with ITP, our previous study have shown that PB Th17 cells were significantly increased, while PB Tregs were numerically decreased and functionally impaired 10. Genotype analysis also indicated that IL-17F 7488 A allele was associated with increased risk of ITP 41. More recently, Song, *et al*. reported that Th17 cells were elevated in parallel with a decrease in Tregs in BM of ITP patients 42. In consistence with these previous reports, our present data showed BM of ITP patients had significantly increased percentage of Th17 cells and remarkably decreased percentage of Tregs. Moreover, levels of the BM Th17 cells were higher, but Tregs were lower than their paired PB counterparts, suggesting a more severe immune dysregulation occurs in BM of ITP patients.

Th1 cells, another subtype of CD4^+^ T cells, have been widely known to be abnormally overactivated in ITP patients 43. Our study observed significantly higher levels of Th1 cells and IFN-γ in BM and PB from patients with ITP, confirming that ITP has a Th1 dominant profile.

It seemed that patients with relative severe bleeding episodes (grade 2 or 3) had higher levels of BM Th1 cells and PB Th22 cells compared to these patients with mild bleeding episodes (grade 1). It might be possible that patients with severe bleeding episodes had a more inflammatory environment. Th22 cells act mostly in skin and mucosal tissues as they express the chemokine receptor CCR6 and the skin homing receptor CCR4 and CCR10 24. In addition, IL-22R1, the receptor subunit of IL-22, was expressed abundantly by barrier surface such as skin, mucosal, and vascular endothelial cells 25, further indicating involvement of Th22 cells in barrier homeostasis regulation. Bleeding symptoms indicated existence of peripheral vascular endothelial damages and inflammation, which might facilitate the chemotaxis of Th22 cells and subsequent wound healing 44. This also might partly explain why frequency of PB Th22 cells was higher from bleeding grade 2 or 3 patients than from bleeding grade 1 patients.

The precise mechanism how Th1, Th17, and Th22 cells accumulated in BM of ITP patients was still unclarified. Migration of these Th cells from PB into BM might be one possible way. CCL27 is a well-known chemoattractant for attracting memory T cells to the sites of skin lesions 45,46. More recently, it has been confirmed that CCR10, the receptor for CCL27, is abundantly expressed in Th22 cells 46. Consequently, the elevation in BM CCL27 could attract more Th22 cells to migrate to BM in ITP patients. OPN is a multifunctional extracellular matrix protein produced by a variety of cells and tissues. It is a major amplifier of the Th1-immune response and has been recognized as a proinflammatory cytokine associated with local inflammation 47. Therefore, increased level of BM OPN could promote the Th1 response, which might be a possible explanation for BM Th1 upregulation in ITP. MIF is a pleiotropic inflammatory mediator which could be secreted by monocytes/macrophages, T- and B-cells as well as endothelia and epithelial cells 48. By counter-regulating glucocorticoid suppression of immune responses and inhibiting activation-induce apoptosis, MIF functions as an essential mediator in T-cell activation 49. Moreover, MIF also exerts a chemokine-like function by promoting migration and recruitment of monocytes and T cells 48. Our observation about MIF elevation might be a reflection of elevated inflammation in BM of ITP patients, and its effect on CD4^+^ T-cell modulation in ITP still needs further investigation.

The relationship between platelet GP-specific autoantibodies and imbalance of CD4^+^ T-cell subsets in ITP remains unclarified. Hu et al. reported circulating Th22 cells were higher in ITP patients who had no detectable anti-GP autoantibodies than those with positive anti-GPIIb/IIIa or anti-GPIb/IX autoantibodies 9. By contrast, our previous studies did not show any correlation between anti-GP autoantibodies and circulating Th17 or Th1 cells in ITP patients 50. Consistently, we did not observe any statistical difference in levels of different BM or PB CD4^+^ T-cell subsets between ITP patients with positive anti-GP autoantibodies and those with negative anti-GP autoantibodies. As autoantibody production involves a complex interaction between antigen presenting cells, T cells, B cells, and platelet autoantigens, the precise role of BM CD4^+^ T-cell subset dysregulation in the disturbance of humoral immune response in ITP still awaits further investigation.

Taking together, the present study demonstrated that ITP had numerically increased numbers of Th22, Th17, Th1, and Tfh cells in parallel with significantly reduced percentage of Tregs in BM, suggesting that the imbalance of CD4^+^ T-cell subsets might be involved in the pathophysiological process of the disease. Although further functional studies are need to clarify the direct influence of these abnormal CD4^+^ T cells on platelet production and destruction, strategies to restore the balance of BM CD4^+^ T-cell subsets might provide therapeutic benefits for ITP patients.

## Supplementary Material

Supplementary figures and tables.

## Figures and Tables

**Table 1:** Demographic and clinical characteristics of ITP patients

Patient No.	Sex/Age (years)	Course of disease (months)	Bleeding symptoms (the SOMG index, bleeding grade)	Platelet count (× 10^9^/L)	Major previous therapy
1	M/62	14	PT (S1, 1)	21	GC
2	M/39	2	EP, PT (S1/M2, 2)	25	None
3	F/73	12	PT (S1, 1)	10	GC, rhTPO, RTX
4	F/20	120	ME, EC (S1/O1, 1)	6	GC, RTX, Danzol
5	F/54	4	GH (M1, 1)	5	None
6	F/50	4	GUH, PT (S1/O2, 2)	7	GC
7	F/48	2	PT (S1, 1)	5	GC, IVIg
8	M/75	4	PT, EC (S1, 1)	10	GC
9	F/73	12	GIH, EC (S1/O3, 3)	9	GC, Danazol
10	F/41	3	PT (S1, 1)	28	GC, IVIg
11	M/46	5	GH, PT (S1/M1, 1)	3	GC
12	M/53	3	PT, EC (S1, 1)	12	GC
13	F/76	9	PT, EC (S1, 1)	19	GC, IVIg
14	F/24	14	CH, PT, EC (S1/M1, 1)	4	GC, rhTPO, RTX
15	M/53	0	PT, EC (S1, 1)	16	None
16	F/30	3	CH, PT (S1/M1, 1)	22	GC, rhTPO
17	F/54	5	GH, PT (S1/M1, 1)	18	GC, rhTPO, IVIg
18	M/59	3	EC (S1, 1)	10	GC
19	F/36	9	PT, EC (S2, 2)	4	GC, rhTPO, CA
20	M/54	60	EC, GH (S1/M3, 3)	24	GC, rhTPO, RTX, DCT
21	F/60	0	PT (S1, 1)	11	None
22	M/56	3	EC (S1, 1)	13	GC
23	F/30	132	PT, ME (S1O2, 2)	38	GC, TPO, DCT
24	M/38	2	PT, EC, GH (S1/M1, 1)	9	None
25	M/58	120	PT (S1, 1)	17	GC, TPO
26	F/29	11	PT, ME (S1O1, 1)	32	GC
27	M/72	180	PT, GH, GIH (S1/M3/O1, 3)	7	GC, IVIg, TPO
**Median**	53	4		14	
**Range**	20-76	0-180		3-28	

PT, petechiae; EP, epistaxis; ME, menorrhagia; EC, ecchymoses; GH, gingival haemorrhage; GUH, genitourinary haemorrhage; GIH, gastrointestinal haemorrhage; CH, conjunctival haemorrhage; GC, glucocorticoid; rhTPO, recombinant human thrombopoietin; RTX, Rituximab; IVIg, intravenous immunoglobulin; CA, caffeic acid; DCT, decitabine.

**Table 2:** CD4^+^ T-cell subsets and their signature cytokines in newly diagnosed/persistent, chronic ITP patients and HCs.

Group Subset	ITPn+p BM (n = 18)	ITPc BM (n = 9)^ §^	ITPn+p PB (n = 18)^&1^	ITPc PB (n = 9)^ &2, §§^	HC BM (n = 15)^ &&1, &&2^	HC PB (n = 15)^ &&&1, &&&2, &&&&^
Th22 (%)	2.22 ± 0.61	2.05 ± 0.96^$^	1.42 ± 0.51^#^	1.45 ± 0.68^#,$^	0.84 ± 0.17^#,#^	0.83 ± 0.16^#,#,$^
IL-22 (pg/ml)	36.94 ± 14.05	38.22 ± 23.63^$^	30.50 ± 11.86^#^	31.50 ± 16.65^#,$^	21.80 ± 2.06^#,#^	20.67 ± 3.49*^,^*^,$^
Th17 (%)	3.37 ± 1.21	3.14 ± 1.00^$^	2.16 ± 0.91^#^	2.17 ± 0.81^#,$^	1.39 ± 0.17^#,#^	1.32 ± 0.22^#,#,$^
Tregs (%)	1.85 ± 0.75	1.71 ± 0.78^$^	3.95 ± 1.11^#^	3.68 ± 1.44^#,$^	6.12 ± 0.30^#,#^	6.21 ± 0.18*^,^*^,$^
Th17/Treg	2.20 ± 1.23	2.17 ± 1.08^$^	0.70 ± 0.68^#^	0.81 ± 0.68^#,$^	0.19 ± 0.03^#,#^	0.18 ± 0.03^#,#,$^
IL-17 (pg/ml)	16.77 ± 2.37	16.12 ± 1.77^$^	16.45 ± 2.46^$^	15.27 ± 2.82^$,$^	13.05 ± 3.27^#,#^	14.77 ± 2.85^$,$,$^
Th1 (%)	25.78 ± 5.78	25.74 ± 7.83^$^	17.45 ± 5.82^#^	18.60 ± 6.00*^,$^	7.70 ± 1.12^#,#^	7.11 ± 1.33^#,#,$^
IFN-γ (pg/ml)	5.45 ± 2.23	5.40 ± 2.29^$^	4.13 ± 1.56^#^	4.08 ± 1.44*^,$^	3.21 ± 0.57^#,#^	3.00 ± 0.31*^,^*^,$^

ITPn+p: newly diagnosed or persistent ITP; ITP_C_: chronic ITP. ^&1^ITPn+p BM *vs*. ITPn+p PB, ^&2^ITPc BM *vs*. ITPc PB, ^&&1^ITPn+p BM *vs*. HC BM, ^&&2^ITPc BM *vs*. HC BM, ^&&&1^ITPn+p PB *vs*. HC PB, ^&&&2^ITPc PB *vs*. HC PB, ^&&&&^HC BM *vs*. HC PB, ^§^ITPn+p BM *vs*. ITP_C_ BM, ^§§^ITPn+p PB *vs*. ITP_C_ PB. *P < 0.05, ^#^*P* < 0.01, ^$^*P >* 0.05.

**Table 3:** CD4^+^ T-cell subsets and their signature cytokines in HCs and ITP patients receiving no/first-line therapy and second-line therapy.

Group Subset	ITP_no/1st-line_ BM (n = 17)	ITP_2nd-line_ BM (n = 10)^ §^	ITP_no/1st-line_ PB (n = 17)^ &1^	ITP_2nd-line_ PB (n = 10)^ &2, §§^	HC BM (n = 15)^ &&1, &&2^	HC PB (n = 15)^ &&&1, &&&2, &&&&^
Th22 (%)	2.12 ± 0.66	2.23 ± 0.86^$^	1.31 ± 0.49^#^	1.64 ± 0.63^#,$^	0.84 ± 0.17^#,#^	0.83 ± 0.16^#,#,$^
IL-22 (pg/ml)	36.73 ± 16.90	38.44 ± 18.99^$^	30.09 ± 12.63^#^	32.09 ± 15.05*^,$^	21.80 ± 2.06^#,#^	20.67 ± 3.49^#,#,$^
Th17 (%)	3.20 ± 1.21	3.44 ± 1.00^$^	2.15 ± 0.95^#^	2.20 ± 0.72^#,$^	1.39 ± 0.17^#,#^	1.32 ± 0.22^#,#,$^
Tregs (%)	1.84 ± 0.62	1.70 ± 0.99^$^	3.94 ± 0.92^#^	3.74 ± 1.64^#,$^	6.12 ± 0.30^#,#^	6.21 ± 0.18^#,#,$^
Th17/Tregs	2.01 ± 1.13	2.74 ± 1.78^$^	0.59 ± 0.36^#^	0.99 ± 0.97^#,$^	0.19 ± 0.03^#,#^	0.18 ± 0.03*^,#,$^
IL-17 (pg/ml)	16.89 ± 2.45	15.98 ± 1.55^$^	15.98 ± 3.19^$^	16.20 ± 1.13^$,$^	13.05 ± 3.27^#,#^	14.77 ± 2.85^$,$,$^
Th1 (%)	25.95± 6.20	25.47 ± 7.00^$^	16.82 ± 3.99^#^	19.53 ± 7.96*^,$^	7.70 ± 1.12^#,#^	7.11 ± 1.33^#,#,*^
IFN-γ (pg/ml)	5.43 ± 2.24	5.45 ± 2.27^$^	3.97 ± 1.50^#^	4.35 ± 1.52*^,$^	3.21 ± 0.57^#,#^	3.00 ± 0.31^#,^*^,$^

ITP_no/1st-line_: ITP patients that has not been treated or used to be treated with first-line drugs; ITP_2nd-line_: ITP patients that used to be treated with second-line drugs. ^&1^ITP_no/1st-line_ BM *vs*. ITP_no/1st-line_ PB, ^&2^ITP_2nd-line_ BM *vs*. ITP_2nd-line_ PB, ^&&1^ITP_no/1st-line_ BM *vs*. HC BM, ^&&2^ITP_2nd-line_ BM *vs*. HC BM, ^&&&1^ITP_no/1st-line_ PB *vs*. HC PB, ^&&&2^ITP_2nd-line_ PB *vs*. HC PB, ^&&&&^HC BM *vs*. HC PB, ^§^ITP_no/1st-line_ BM *vs*. ITP_2nd-line_ BM, ^§§^ITP_no/1st-line_ PB *vs*. ITP_2nd-line_ PB. *P < 0.05, ^#^*P* < 0.01, ^$^*P >* 0.05.

**Table 4:** CD4^+^ T-cell subsets and their signature cytokines in treatment-naïve ITP patients, recurrent ITP patients, and HCs.

Group Subset	ITP_r_ BM (n = 22)	ITP_tn_ BM c (n = 5)^ §^	ITP_r_ PB (n = 22)^ &1^	ITP_tn_ PB (n = 5)^ &2, §§^	HC BM (n = 15)^ &&1, &&2^	HC PB (n = 15)^ &&&1, &&&2, &&&&^
Th22 (%)	2.19 ± 0.74	2.05 ± 0.71^$^	1.46 ± 0.59^#^	1.29 ± 0.38*^,$^	0.84 ± 0.17^#,#^	0.83 ± 0.16^#,$,$^
IL-22 (pg/ml)	38.29 ± 17.21	33.31 ± 19.46^$^	31.92 ± 13.61^#^	20.04 ± 12.12^$,$^	21.80 ± 2.06^#,$^	20.67 ± 3.49^#,$,$^
Th17 (%)	3.39 ± 1.21	2.84 ± 0.45^$^	2.24 ± 0.86^#^	1.86 ± 0.87*^,$^	1.39 ± 0.17^#,#^	1.32 ± 0.22^#,$,$^
Tregs (%)	1.78 ± 0.83	1.83 ± 0.43^$^	3.85 ± 1.25^#^	3.93 ± 1.11^#,$^	6.12 ± 0.30^#,#^	6.21 ± 0.18^#,#,$^
Th17/Treg	2.42 ± 1.52	2.68 ± 0.69^$^	0.77 ± 0.70^#^	0.59 ± 0.56^#,$^	0.19 ± 0.03^#,*^	0.18 ± 0.03^#,$,$^
IL-17 (pg/ml)	16.88 ± 2.21	15.11 ± 1.38^$^	15.91 ± 2.80^$^	16.69 ± 1.39^$,$^	13.05 ± 3.27^#,$^	14.77 ± 2.85^$,$,$^
Th1 (%)	25.32 ± 6.33	27.75 ± 6.97^$^	17.68 ± 5.10^#^	18.50 ± 8.99*^,$^	7.70 ± 1.12^#,#^	7.11 ± 1.33^#,#,$^
IFN-γ (pg/ml)	5.41 ± 2.09	5.55± 2.94^$^	4.08 ± 1.43^#^	3.87 ± 1.79*^,$^	3.21 ± 0.57^#,*^	3.00 ± 0.31*^,$,$^

ITP_r_: recurrent ITP; ITP_tn_: treatment-naive ITP. ^&1^ITP_r_ BM *vs*. ITP_r_ PB, ^&2^ITP_tn_ BM *vs*. ITP_tn_ PB, ^&&1^ITP_r_ BM *vs*. HC BM, ^&&2^ITP_tn_ BM *vs*. HC BM, ^&&&1^ITP_r_ PB *vs*. HC PB, ^&&&2^ITP_tn_ PB *vs*. HC PB, ^&&&&^HC BM *vs*. HC PB, ^§^ITP_r_ BM *vs*. ITP_tn_ BM, ^§§^ITP_r_ PB *vs*. ITP_tn_ PB. *P < 0.05, ^#^*P* < 0.01, ^$^*P >* 0.05.

**Table 5:** CD4^+^ T-cell subsets in bleeding grade 1 patients, bleeding grade 2 or 3 patients, and HCs.

Group Subset	ITP_grade 1_ BM (n = 20)	ITP_grade 2+3_ BM (n = 7)^§^	ITP_grade 1_ PB (n = 20)	ITP_grade 2+3_ PB (n = 7)^§§^	HC BM (n = 20)^&&1, &&2^	HC PB (n = 7)^&&&1, &&&2^
Th22 (%)	2.03 ± 0.72	2.54 ± 0.65^$^	1.31 ± 0.53	1.78 ± 0.50*	0.84 ± 0.17^#,#^	0.83 ± 0.16^#,#^
Th17 (%)	3.26 ± 1.16	3.36 ± 1.10^$^	2.10 ± 0.92	2.36 ± 0.66^$^	1.39 ± 0.17^#,#^	1.32 ± 0.22^#,#^
Tregs (%)	1.89 ± 0.82	1.49 ± 0.48^$^	4.00 ± 1.21	3.47 ± 1.20^$^	6.12 ± 0.30^#,#^	6.21 ± 0.18^#,#^
Th17/Tregs	2.15 ± 1.29	2.63 ± 1.81^$^	0.70 ± 0.69	0.84 ± 0.64^$^	0.19 ± 0.03^#,#^	0.18 ± 0.03^#,^*
Th1 (%)	24.19± 6.00	30.28 ± 6.72^#^	17.37 ± 5.02	19.13 ± 7.94^$^	7.70 ± 1.12^#,#^	7.11 ± 1.33^#,#^

ITP_grade 1_: Bleeding grade 1 patients; ITP_grade 2+3_: Bleeding grade 2 or 3 patients. ^&&1^ITP_grade 1_ BM *vs*. HC BM, ^&&2^ITP_grade 2+3_ BM *vs*. HC BM, ^&&&1^ITP_grade 1_ PB *vs*. HC PB, ^&&&2^ITP_grade 2+3_ PB *vs*. HC PB, ^§^ITP_grade 1_ BM *vs*. ITP_grade 2+3_ BM, ^§§^ITP_grade 1_ PB *vs*. ITP_grade 2+3_ PB. *P < 0.05, ^#^*P* < 0.01, ^$^*P >* 0.05.

**Table 6:** Chemokine receptors expressed by different CD4^+^ T-cell subsets in BM and PB of ITP patients and HCs.

Group Chemokine receptor	ITP BM n = 6	ITP PB^&^ n = 6	HC BM^&&^ n = 6	HC PB^&&&, &&&&^ n = 6
CXCR3 on CD4^+^IFN-γ^+^	1527.3 ± 216.1	1705.5 ± 235.1*	1063.5 ± 217.5^#^	1293.7 ± 141.6^#, #^
CCR4 on CD4^+^IL-4^+^	16666.3 ± 7164.2	17100.3 ± 8186.8^$^	13363.7 ± 539.1^$^	13589.7 ± 604.7^$, $^
CCR4 on CD4^+^IL-17^+^	1160.7 ± 393.8	1047.5 ± 524.8^$^	1375.2 ± 166.9^$^	1173.3 ± 282.8^$, $^
CCR6 on CD4^+^IL-17^+^	1259.2 ± 273.2	1350.3 ± 395.9^$^	1508.2 ± 141.0^$^	1379.2 ± 127.0^$, $^
CCR4 on CD4^+^IL-22^+^	2584.3 ± 824.5	2402.8 ± 607.3^$^	1624.8 ± 217.4^#^	1496.2 ± 155.4^#, $^
CCR10 on CD4^+^IL-22^+^	1673.0 ± 357.3	1666.3 ± 364.8^$^	1495.5 ± 106.3^$^	1514.3 ± 105.5^$, $^
CXCR4 on CD4^+^FoxP3^+^	1928.8 ± 154.3	1861.5 ± 240.2^$^	1752.0 ± 112.3^$^	1752.3 ± 128.5^$, $^

^&^ITP BM *vs*. ITP PB, ^&&^ITP BM *vs*. HC BM, ^&&&^ITP PB *vs*. HC PB, ^&&&&^HC BM *vs*. HC PB; *P < 0.05, ^#^*P* < 0.01, ^$^*P >* 0.05.

## Abbreviations

ITP: primary immune thrombocytopenia
PB: peripheral blood
BM: bone marrow
Th: T helper
Tfh: follicular T helper
Tregs: regulatory T cells
IL: interleukin
ELISA: enzyme-linked immunosorbent assay
CTL: cytotoxic T lymphocyte
MK: megakaryocyte
MDS: myelodysplasia syndrome
AA: aplastic anemia
ITP-BAT: ITP-specific Bleeding Assessment Tool
HC: healthy control
RPMI: Roswell Park Memorial Institute
PMA: phorbol myristate acetate
PE: phycoerythrin
mAbs: monoclonal antibodies
FITC: fluorescein isothiocyanate
APC: allophycocyanin
MFI: median fluorescence intensity
MAIPA: mAb-specific immobilization of platelet antigens
GP: glycoprotein
AICD: activation induced cell death
TNF: tumor necrosis factor
SLE: systemic lupus erythematosus
RA: rheumatoid arthritis
TGF: transform growth factor
OPN: osteopontin
