# Supplementary material for: Disrupted balance of CD4+ T-cell subsets in bone marrow of patients with primary immune thrombocytopenia
Source: Int J Biol Sci. 2019 Oct 23;15(13):2798–814. doi: 10.7150/ijbs.33779 (PMC6909963; doi:10.7150/ijbs.33779)

Disrupted balance of $CD4^+$ T-cell subsets in bone marrow of patients with primary immune thrombocytopenia

Supplemental data

Supplemental methods

Immunofluorescence staining of $CD4^+$ T-cell subsets

As it was difficult to conduct T-cell stimulation and intracellular cytokine staining on BM smear slides, a crucial process for determination of Th1, Th2, Th17 cells, and Tregs which were mainly identified by intracellular protein markers, we stimulated and stained $CD4^+$ T-cell subsets first, and prepared slides by the squash method for immunofluorescence analysis. Briefly, bone marrow mononuclear cells (BMMCs) were obtained from heparinized BM blood by gradient centrifugation on Ficoll-Paque, stimulated with 25 ng/ml phorbol myristate acetate (PMA), 1 μg/ml ionomycin, and 1.7 μg/ml Golgiplug at 37℃ for 4 hours, washed, and incubated with APC-conjugated anti-CD4 mAbs (eBioscience). The cells were next stained with FITC-conjugated anti-IFN-γ mAbs (eBioscience) after permeabilization, and resuspended for Th1 analysis.

For Treg staining, BMMCs were incubated with FITC-conjugated anti-CD4 mAbs (eBioscience), fixed and permeabilized, then stained with APC-conjugated anti-FoxP3 mAbs (eBioscience), washed, and finally resuspended for Treg analysis.

For determination of $CD4^+$ T-cell subsets by chemokine receptors, BMMCs were stained with APC-conjugated anti-CD4 mAbs and FITC-conjugated anti-CXCR3 mAbs (Biolegend) for Th1 analysis. FITC-conjugated anti-CD4 mAbs, Alexa Flour 647-conjugated anti-CCR4 mAbs (Biolegend) and PE-conjugated anti-CCR6 (Biolegend) were incubated with BMMCs for Th17 and Th2 identification, while FITC-conjugated anti-CD4 mAbs and APC-conjugated anti-CCR10 mAbs (Biolegend) were used for Th22 analysis.

The cell suspension was dropped on the slides which were next mounted in fluoroshield mounting medium with DAPI (Abcam) to stain the nuclear and prevent fluorescence

self-quenching. The slides were covered and placed in the fluorescence microscope (Invitrogen EVOS™ FL Auto 2) for $CD4^+$ T-cell subset observation.

Supplemental Table 1. Quantibody® Human Chemokine Array 1 (40) Antibody List

6Ckine/CCL21	GCP-2/CXCL6	IL-29/IFN lambda1	MCP-3/CCL7	NAP-2/CXCL7
Axl/tyro7	GRO(CXCL1,2&3)	IL-31	MCP-4/CCL13	Osteopontin (OPN)
Betacellulin (BTC)	HCC-1/CCL14	IP-10/CXCL10	MDC/CCL22	PARC/CCL18
CCL28	HCC-4/CCL16	I-TAC/CXCL11	MIF	PF4/CXCL4
CTACK/CCL27	IL-9	LIF	MIP-3 alpha/CCL20	SDF-1 alpha/CXCL12a
CXCL16	IL-17F	LIGHT/TNFSF14	MIP-3 beta/CCL19	TARC/CCL17
ENA-78/CXCL5	IL-18 Bpa	Lymphotactin/XCL1	MPIF-1/CCL23	TECK/CCL25
Eotaxin-3/CCL26	IL-28A	MCP-2/CCL8	MSP (alpha chain)	TSLP

Supplemental Table 2. CD4$^+$ T-cell subsets in anti-GP autoantibody positive or negative patients

Group / Subset		anti-GPIIb/IIIa or anti-GPIb/IX (+), n = 15	anti-GPIIb/IIIa and anti-GPIb/IX (-), n = 12	*P*
BM	Th22 (%)	1.98 ± 0.83	2.39 ± 0.52	0.137
	Th17 (%)	3.12 ± 1.19	3.50 ± 1.05	0.395
	Tregs (%)	1.81 ± 0.68	1.76 ± 0.89	0.857
	Th1 (%)	25.30 ± 6.70	26.35 ± 6.19	0.678
PB	Th22 (%)	1.33 ± 0.58	1.56 ± 0.52	0.275
	Th17 (%)	2.06 ± 1.00	2.30 ± 0.67	0.455
	Tregs (%)	4.13 ± 0.84	3.53 ± 1.53	0.241
	Th1 (%)	16.59 ± 5.82	19.38 ± 5.61	0.220

Supplemental Figure legends

Supplemental Figure 1. Th22, Th17, Th1, Th2, and Tregs in BM smear were visualized by immunofluorescence. (A, B) $CD4^+$ (green) $CCR10^+$ (red) T cells (Th22 cells) in ITP patients and HCs. **(C, D)** $CD4^+$ (green) $CCR4^+$ (red) $CCR6^+$ (orange) T cells (Th17 cells) and $CD4^+$ (green) $CCR4^+$ (red) $CCR6^-$ T cells (Th2 cells) in ITP patients and HCs. **(E, F)** $CD4^+$ (red) $CXCR3^+$ (green) T cells (Th1 cells) in ITP patients and HCs. **(G, H)** $CD4^+$ (red) IFN-γ^+ (green) T cells (Th1 cells) in ITP patients and HCs. **(I, J)** $CD4^+$ (green) $FoxP3^+$ (red) T cells (Tregs) in ITP patients and HCs.

Supplemental Figure 2. Percentages of BM Th22, Th17, Th1, Th2, and Tregs quantified by immunofluorescence assay. (A, B) Percentages of BM Th22 and Th17 cells were slightly higher from ITP patients compared with HCs but statistical significance was not achieved. **(C, D)** Percentage of BM Tregs was significantly lower, whereas Th1 frequency was remarkably higher from ITP patients in comparison with these from HCs. (E) There was no statistical difference in BM Th2 cells between ITP patients and HCs. Bars represent SD. * $P < 0.05$.

Supplemental Figure 1.

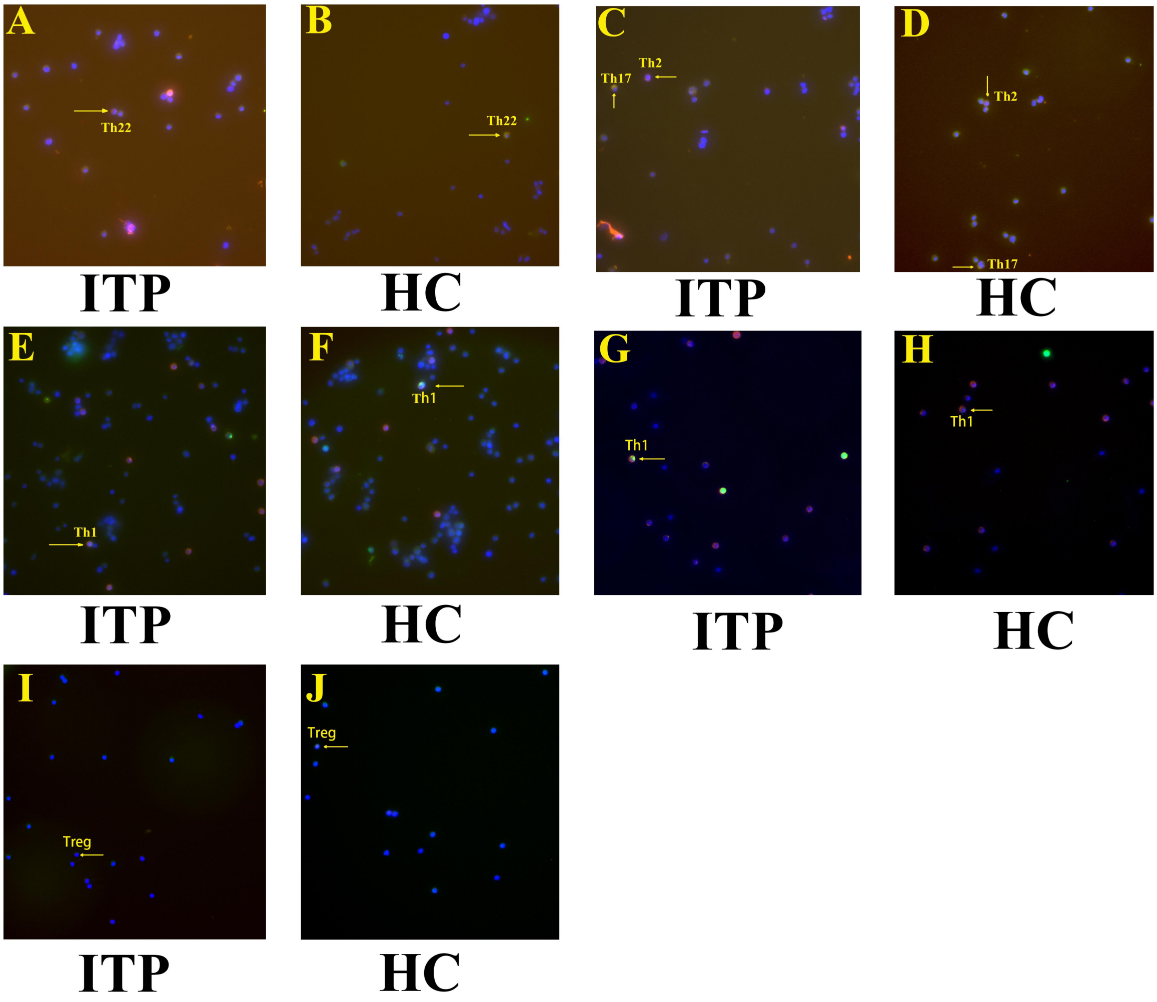

Supplemental Figure 2.

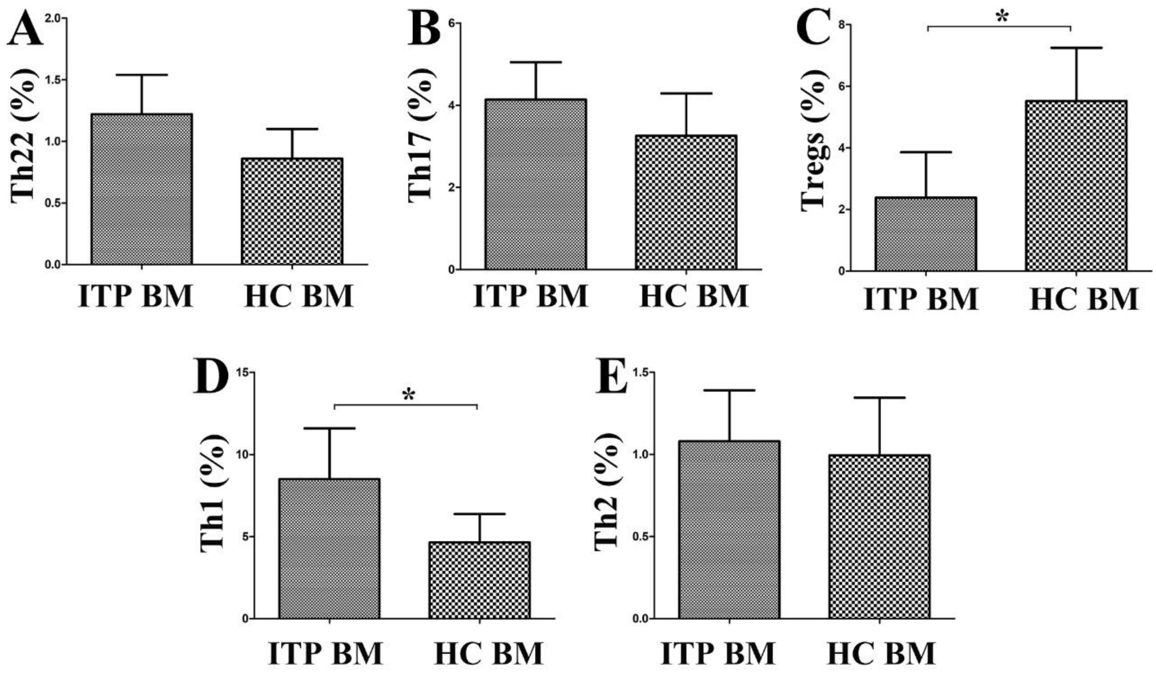

Supplement: Supplementary file 1 — Supplementary figures and tables. [file ijbsv15p2798s1.pdf]
